# Pleiotropic Effects of *Blastocystis* spp. Subtypes 4 and 7 on Ligand-Specific Toll-Like Receptor Signaling and NF-κB Activation in a Human Monocyte Cell Line

**DOI:** 10.1371/journal.pone.0089036

**Published:** 2014-02-14

**Authors:** Joshua D. W. Teo, Paul A. MacAry, Kevin S. W. Tan

**Affiliations:** 1 Laboratory of Molecular and Cellular Parasitology, Department of Microbiology, Yong Loo Lin School of Medicine, National University of Singapore, Singapore, Singapore; 2 Immunology Program, National University of Singapore, Singapore, Singapore; SRI International, United States of America

## Abstract

*Blastocystis* spp. is a common enteric stramenopile parasite that colonizes the colon of hosts of a diverse array of species, including humans. It has been shown to compromise intestinal epithelial cell barrier integrity and mediate the production of pro-inflammatory cytokines and chemokines. Mucosal epithelial surfaces, including the intestinal epithelium, are increasingly recognized to perform a vital surveillance role in the context of innate immunity, through the expression of pathogen recognition receptors, such as Toll-like receptors (TLRs). In this study, we use the human TLR reporter monocytic cell line, THP1-Blue, which expresses all human TLRs, to investigate effects of *Blastocystis* on TLR activation, more specifically the activation of TLR-2, -4 and -5. We have observed that live *Blastocystis* spp. parasites and whole cell lysate (WCL) alone do not activate TLRs in THP1-Blue. Live ST4-WR1 parasites inhibited LPS-mediated NF-κB activation in THP1-Blue. In contrast, ST7-B WCL and ST4-WR1 WCL induced pleiotropic modulation of ligand-specific TLR-2 and TLR-4 activation, with no significant effects on flagellin-mediated TLR-5 activation. Real time-qPCR analysis on SEAP reporter gene confirmed the augmenting effect of ST7-B on LPS-mediated NF-κB activation in THP1-Blue. Taken together, this is the first study to characterize interactions between *Blastocystis* spp. and host TLR activation using an in vitro reporter model.

## Introduction

*Blastocystis* is an enteric protistan parasite that colonizes the colonic epithelia of human and animal hosts and is phylogenetically classified among the Stramenopiles [1], [2]. Due to its low species specificity and the highly zoonotic nature of numerous isolates of the parasite, previous species-naming conventions have become less favored and the parasite is termed as the species complex *Blastocystis* spp. Identifying nomenclature of the parasite has consolidated into a system of consensus terminology [3], which classifies all known *Blastocystis* spp. isolates into subtypes based on sequence similarities in small-subunit ribosomal RNA. To date, 17 subtypes have been identified from mammalian and avian hosts alone, of which 9 are found in humans [4]. Most carriers of *Blastocystis* spp. remain asymptomatic, especially those of subtype 2 [5]; with typical gastrointestinal symptoms associated with *Blastocystis* spp. infections comprising of diarrhea, abdominal pain, flatulence, vomiting and bloating [6]. The parasite has also been implicated in allergy-associated dermatological conditions and irritable bowel syndrome [7], [8] (IBS).

Recent findings have begun to provide a better understanding of the pathogenesis of *Blastocystis* spp. and to confirm its status as an emerging pathogen. Earlier studies reported that some isolates of *Blastocystis* spp. are capable of degrading human secretory immunoglobulin A [9]; and promote contact-independent apoptosis, F-actin rearrangement and barrier function disruption in a non-transformed rat intestinal epithelial cell line [10]. Most recently, cysteine proteases of the parasite are reported to induce rho kinase-mediated intestinal epithelial barrier compromise in human colonic epithelial cells [11]. Despite these reports, much still remains to be understood about interactions between *Blastocystis* spp., host colonic epithelia and associated gut mucosal immunity.

Toll-like receptors (TLRs) are a family of pathogen recognition receptors that play a vital role in innate immune-surveillance of microbial molecular patterns. All TLRs share three similar structural features: a divergent ligand-binding extracellular domain with leucine-rich repeats, a short transmembrane region, and a highly homologous cytoplasmic toll/interleukin (IL)-1 receptor domain that is similar to that of the IL-1 receptor family and is essential for initiation of downstream signaling cascades [12]. 13 TLRs have been identified in humans and mice, seven of which are expressed in the intestinal mucosa [13].

TLRs in the intestinal mucosa are able to rapidly recognize luminal pathogens and their associated molecular patterns, while still maintaining hyporesponsiveness to persistently present populations of harmless commensals [12]. Upon activation by pathogen-associated factors, TLRs activate downstream signaling cascades which mediate the activation of transcription factors such as NF-κB and culminate in the upregulation of immune response genes, such as those of pro-inflammatory cytokines and chemoattractant chemokines to facilitate immune cell infiltration to the site of infection. Recent studies also suggest possible immune-regulatory roles of TLRs towards maintenance of intestinal homeostasis by regulating barrier function and modulating mucosal immune response; and management of intestinal injury through promoting the proliferation of intestinal epithelial cells [13]. We hypothesize that given the various effects of *Blastocystis* spp. on host intestinal epithelial cells, it is likely that the parasite would have a dysregulating effect on TLR signaling, contributing towards a disruption of intestinal homeostasis and the manifestation of gastrointestinal disease states.

It is currently unknown if *Blastocystis* spp. activates TLRs or has any modulating effects on TLR signaling. In the current study, we employed THP1-Blue, a commercially available human monocytic TLR reporter cell line to investigate *Blastocystis* effects on TLR activation. The THP1 human monocytic cell line naturally express many pattern recognition receptors, including TLRs. THP1-Blue cells are modified from this cell line via a stable transfection of a reporter plasmid that expresses a secreted embryonic alkaline phosphatase (SEAP) gene under the control of a promoter inducible by NF-κB. By studying the interactions of *Blastocystis* spp. on TLRs relevant to gut mucosa, we aim to use this approach to obtain preliminary insights into *Blastocystis* spp. effects on TLR signaling.

## Materials and Methods

### Culture of THP1-Blue Human Monocytic Cell Line

In this study, the THP1-Blue human monocytic cell line (Invivogen) was used for all experiments investigating *Blastocystis*-host interactions. THP1-Blue cells were maintained in T-75 flasks in a humidified incubator at 37°C and 5% CO_2_. The THP1-Blue cells were cultured in Roswell Park Memorial Institute (RPMI) 1640 (Gibco) supplemented with 100 U/ml penicillin and 100 µg/ml streptomycin (PAN-Biotech) and 10% heat-inactivated fetal bovine serum (Gibco). Culture viability was evaluated every 3–4 days using the trypan blue assay and only cultures with >95% viability were used for experiments.

### Culture of *Blastocystis* Parasites and Whole Cell Lysate

In this study, 2 axenic isolates from 2 subtypes of *Blastocystis* spp. were used. According to the consensus classification terminology proposed by Stensvold [3], they are *Blastocystis* spp. subtype 4-isolate WR1 (ST4-WR1) and subtype 7-isolate B (ST7-B). ST7-B was originally isolated from a patient with abdominal symptoms at Singapore General Hospital and maintained as an axenic culture in our laboratory [9], [14]. ST4-WR1 was isolated from asymptomatic laboratory rodents in our laboratory and subsequently axenized [9], [15]. Both ST4-WR1 and ST7-B were maintained in pre-reduced Iscove’s modified Dulbecco’s medium (IMDM) (Hyclone), supplemented with 10% heat-inactivated horse serum (Gibco). The culture tubes were cultured in anaerobic jars (Thermo Scientific-Oxoid). Parasites were sub-cultured every 3–4 days. 24 hour old cultures were used directly for experiments involving live parasites, for parasite WCL preparation. For preparation of WCL, 24 hour old parasites were collected from culture tubes and washed twice in sterile phosphate-buffered saline (PBS) (pH 7.4). Parasites were then counted using a hemocytometer and subjected to 3 freeze-thaw cycles in liquid nitrogen and 37°C water bath. WCL aliquots were stored at −80°C before use.

### SEAP Activity Assay

**Figure 1:**
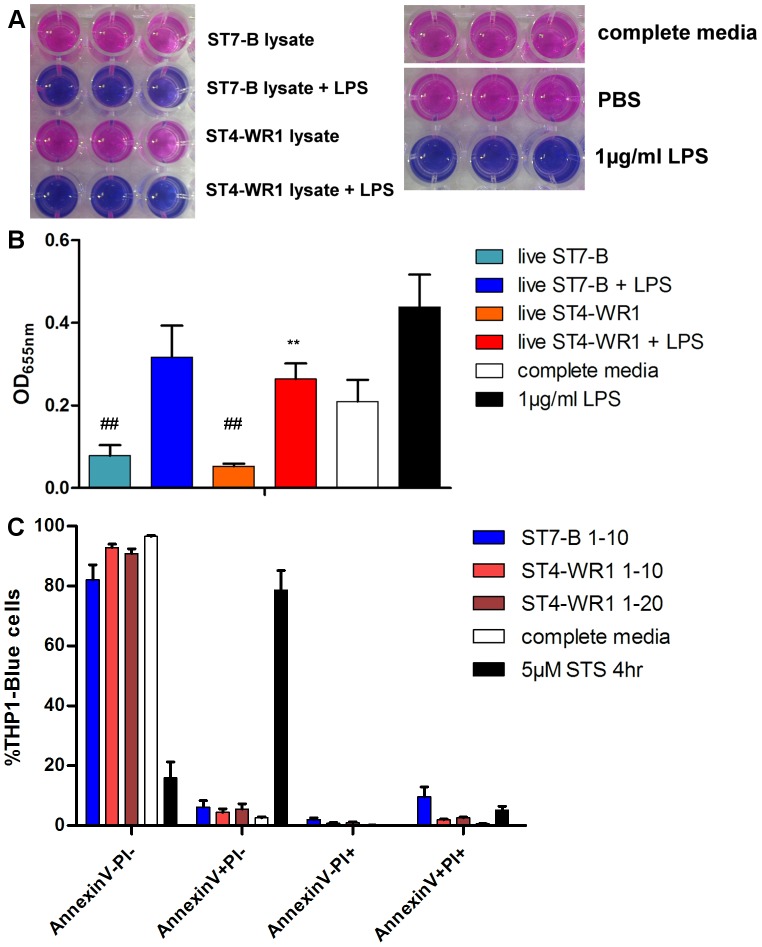
Effects of live *Blastocystis* on LPS stimulation of THP1-Blue monocytes. A. Representative images of colorimetric changes to THP1-Blue cell culture supernatant, indicating SEAP activity of substrate medium. B. Inter-isolate variation in live *Blastocystis* in modulating effects on LPS stimulation of THP1-Blue monocytes. Detection of SEAP activity from cell culture supernatants of THP1-Blue monocytes incubated with various conditions. Live parasites incubated with THP1-Blue cells in numbers corresponding to ratio of 1 THP1-Blue cell to 10 parasites (1–10), or 1 THP1-Blue cell to 20 parasites (1–20). Live ST4-WR1 and not ST7-B significantly dampens LPS-mediated NF-κB activation in THP1-Blue monocytes. Live parasites of both isolates also significantly reduce background absorbance. C. AnnexinV-FITC PI viability assessment of THP1-Blue monocytes after exposure to live parasites. Slight but consistent decrease in viability in THP1-Blue monocytes after exposure to live parasites of both isolates. **, *p*<0.01, when analyzed against 1 µg/ml LPS positive control. ##, *p*<0.01, when analyzed against complete media negative control.

In this study, unless otherwise stated, THP1-Blue cells were exposed to live parasites and parasite WCL at the ratio of 1 THP1-Blue cell to 10–20 parasites for 24 hours under THP1-Blue culture conditions (37°C and 5% CO_2_). 1 µg/ml purified lipopolysaccharide (LPS) from *Salmonella enterica* serotype *typhimurium* (Sigma Aldrich), 5 µg/ml purified zymosan (ZG) from *Saccharomyces cerevisiae* (Invivogen) or 0.5 µg/ml purified flagellin (fla) from *Salmonella enterica* serotype *typhimurium* (Invivogen) were used in conjunction with live parasites or parasite WCL in co-culture conditions with THP1-Blue cells. Culture supernatant was collected and incubated with QUANTI-Blue substrate medium for 2 hours at 37°C. SEAP activity was then assessed by colorimetric change (figure 1A) in the substrate medium and absorbance reading at 655 nm with a Infinite M200 microplate reader (Tecan).

### THP1-Blue Cell Viability Assay

Viability of THP1-Blue cells was studied by annexin V-fluorescein isothiocyanate (FITC)-propidium iodide (PI) staining with an Annexin V-FITC Apoptosis Detection Kit (Biovision), following closely the manufacturer’s protocol recommendations. 5 µM staurosporine (STS) was used as a positive control to induce cell death. Briefly, THP1-Blue cells were washed and resuspended in annexin V binding buffer from the kit after exposure to various experimental conditions described above. FITC-labeled annexin V and PI were then added to the cell suspension. Stained THP1-Blue cells were then subjected to flow cytometric analysis using THP1-Blue cells were differentiated from live parasites due to exclusion by size gating on non-overlapping populations on forward scatter-side scatter dot plots during flow cytometric analysis. Offline flow cytometric analysis was carried out using FlowJo Ver. 7.6.4.

### Real-time Quantitative PCR

After exposure to parasite lysate, total RNA was extracted from THP1-Blue cells using the RNeasy Mini Kit (Qiagen). 1 µg total RNA was reverse-transcribed using iScript Reverse Transcription Supermix for RT-qPCR (Bio-Rad). Reverse transcription reactions were incubated at 25°C for 5 min, 42°C for 30 min and lastly, 85°C for 5 min. Real-time quantitative PCR was run in Bio-Rad iQ5 Multicolor Real-Time PCR Detection System (Bio-Rad) with iTaq Universal SYBR® Green Supermix (Bio-Rad). The real-time quantitative PCR primer used to target the SEAP gene is as follows: forward, 5′-AGAACCTCATCATCTTCCTG-3′; reverse, 5′-TCCTTCTTCTGCCCTTTTAG-3′. Messenger RNA (mRNA) levels were normalized against glyceraldehyde-3-phosphate dehydrogenase (GADPH).

### Statistical Analysis

Experiments were repeated independently at least twice. Experimental data are analyzed using Student’s t test. A P value of <0.05 is considered statistically significant.

## Results

### Effects of Live ST7-B and ST4-WR1 Parasites on NF-κB Activation in THP1-Blue Cells

Live ST7-B and ST4-WR1 parasites were incubated with THP1-Blue cells and assayed for SEAP secretion, which is a measure of NF-κB activation. SEAP activities in culture supernatant taken from THP1-Blue cells exposed to parasites of either isolate were measured to be markedly lower than background values taken from the complete media negative control (ST7-B, *p*<0.01; ST4-WR1, *p*<0.01) (figure 1B). This suggests that parasites of both isolates significantly reduce background NF-κB activation in THP1-blue cells. When incubated concurrently with TLR-4 ligand LPS, live parasites of both isolates reduced SEAP activity, indicating a dampening of NF-κB activation. However, only ST4-WR1-mediated SEAP activity reduction is significantly lower than that observed in the 1 µg/ml LPS positive control (*p*<0.01). To exclude the possibility that *Blastocystis* spp. was inducing cell death in THP1-Blue, viability of the THP1-Blue cells after exposure to live parasites was assessed by flow cytometry analysis of AnnexinV-FITC, PI staining. Viability of THP1-Blue cells was found to decrease slightly [81.55–92.74%] after being exposed to live ST7-B and ST4-WR1 parasites (figure 1C). This slight decrease in cell viability may explain in part the reduction in background THP1-Blue NF-κB activation. To investigate effects of live ST7-B and ST4-WR1 parasites on SEAP produced from activated THP1-Blue cells, conditioned culture supernatant from THP1-Blue cells that had prior exposure to LPS was also incubated with live ST7-B and ST4-WR1 parasites. SEAP activity in these culture supernatant samples was then measured. SEAP activity was found to be decreased in culture supernatant samples that were incubated with live ST7-B and ST4-WR1 parasites (data not shown). This suggests that live ST7-B and ST4-WR1 parasites were able to inhibit the activity of SEAP produced from activated THP1-Blue cells after ligand stimulation. This, taken together with the previously observed slight decrease in THP1-Blue cell viability, may explain the observations reported in figure 1B.

**Figure 2:**
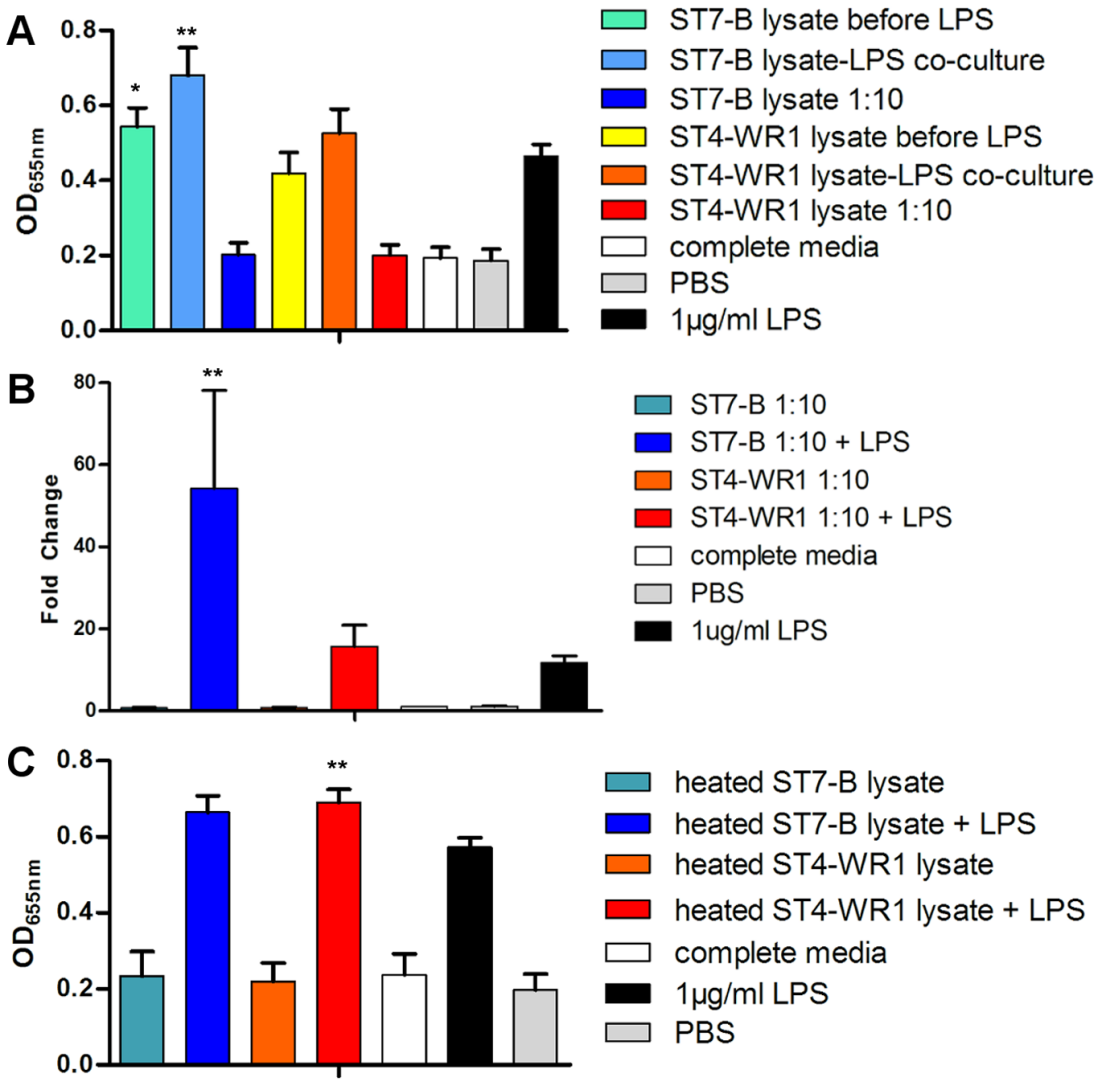
Effects of *Blastocystis* lysate on LPS stimulation of THP1-Blue monocytes. A. Detection of SEAP activity from cell culture supernatants of THP1-Blue monocytes incubated with various conditions. *Blastocystis* total cell lysate incubated with THP1-Blue cells at amounts corresponding to ratio of 1 monocyte to 10 parasites. *Blastocystis* ST7-B, not ST4-WR1, significantly augments LPS-induced activation of THP1-Blue monocytes, as observed in the increased SEAP activity. B. RT-qPCR data of SEAP gene in THP1-Blue cells. Significant increase in fold change of SEAP gene expression observed only in THP1-Blue cells co-cultured with ST7-B WCL and LPS. C. Heat-inactivated lysate of both ST7-B and ST4-WR1 shows augmenting effect on LPS-induced THP1-Blue activation. **, *p*<0.01; *, *p*<0.05, when analyzed against 1 µg/ml LPS positive control.

WCL of both isolates was incubated with THP1-Blue cells and similarly assayed for SEAP secretion. Previously observed effects of live parasites on THP1-Blue, in the presence or absence of LPS, was not observed with whole *Blastocystis* lysate. WCL of both isolates did not have any significant effect on background SEAP activity when compared to the complete media negative control. Interestingly, augmentation of SEAP activity in the presence of LPS was observed only with ST7-B WCL and not ST4-WR1 WCL. This increased SEAP activity was significant for both THP1-Blue cells pre-treated with ST7-B WCL before LPS exposure and cells co-cultured with both ST7-B WCL and LPS (*p*<0.05; *p*<0.01 respectively) (figure 2A).

To verify that observed increase in SEAP activity is attributed to an increase in expression of the SEAP gene, reverse transcription real time-PCR was carried out to assess expression levels of the SEAP gene in THP1-Blue cells after exposure to different experimental conditions (figure 2B). Significant increase in fold change of SEAP mRNA transcription levels was observed only in THP1-Blue cells co-cultured in the presence of both ST7-B WCL and LPS (*p*<0.01). This is consistent with the SEAP enzymatic activity observations described in figure 2A.

### Heat-stable Components in ST7-B WCL Augment LPS-induced NF-κB Activation

WCL of both isolates was also heat-treated at 95°C for 10 minutes before incubating with THP1-Blue cells, in the presence or absence of LPS, for 24 hours. (figure 2C) THP1-Blue cells did not show significantly different levels of SEAP activity when exposed to heated *Blastocystis* WCL, compared to the complete media negative control. There was also no significant deviation from the previously observed increase in SEAP activity in the presence of LPS and ST7-B WCL. Interestingly, Heat treatment of ST4-WR1 WCL was observed to augment SEAP activity in the presence of LPS, to levels similar to those observed for heated ST7-B WCL.

### Varied Effects of ST7-B WCL on NF-κB Activation by other TLR Ligands

**Figure 3:**
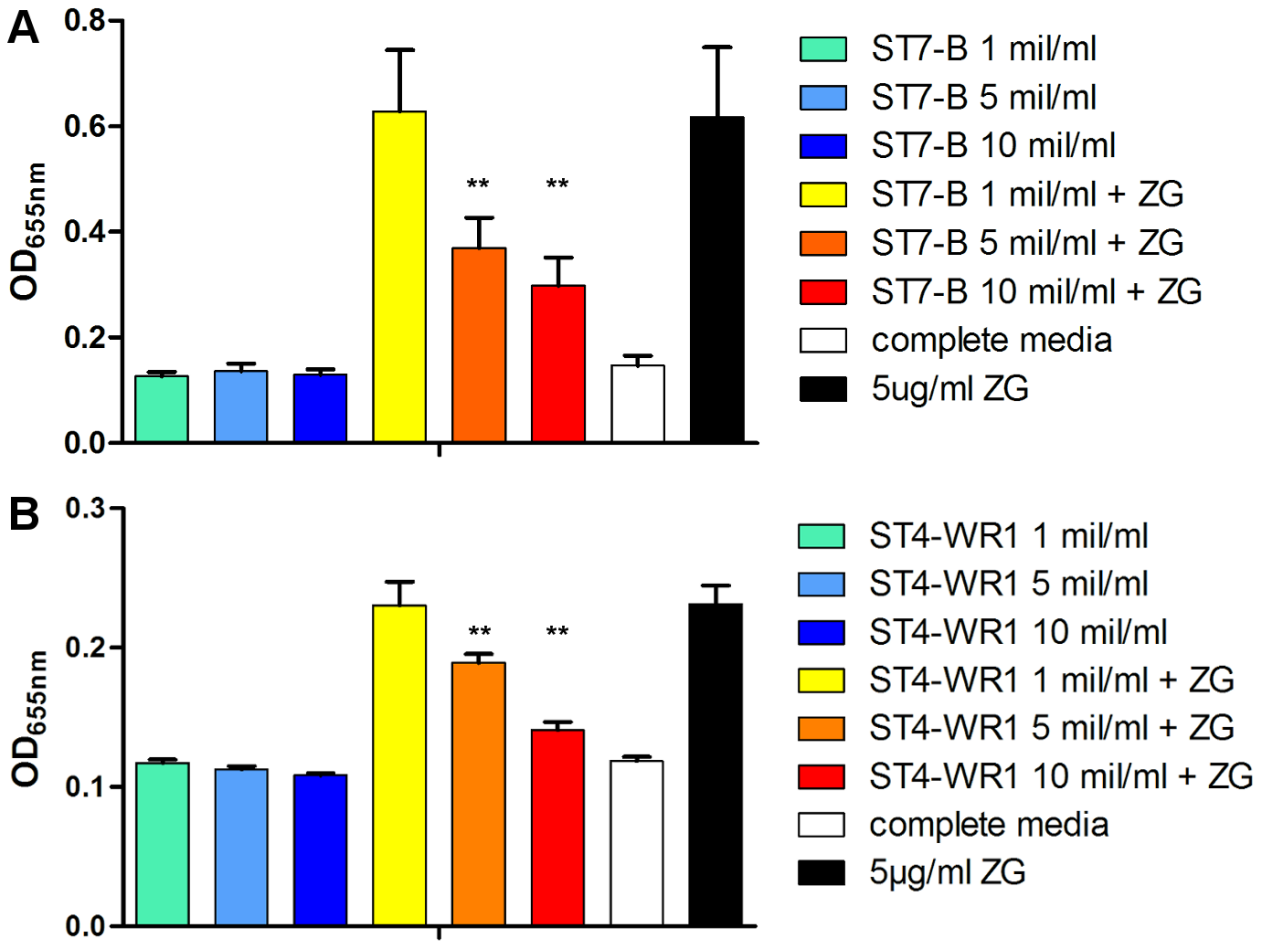
*Blastocystis* effects on zymosan (ZG) stimulation of THP1-Blue monocytes. Detection of SEAP activity from cell culture supernatants of THP1-Blue monocytes incubated with various conditions. A, B. Both *Blastocystis* ST7-B and ST4-WR1 significantly inhibits ZG-induced activation of THP1-Blue monocytes, as observed in the decrease in SEAP activity. Parasite lysate was added to THP1-Blue cells in terms of amounts of lysate prepared from 1 million (mil), 5 million, or 10 million parasites per ml. Inhibition observed to be in negative correlation with parasite lysate concentration, with significance observed from 5×10^6^ ST7-B/ml onward. **, *p*<0.01, when analyzed against 5 µg/ml ZG positive control.

**Figure 4:**
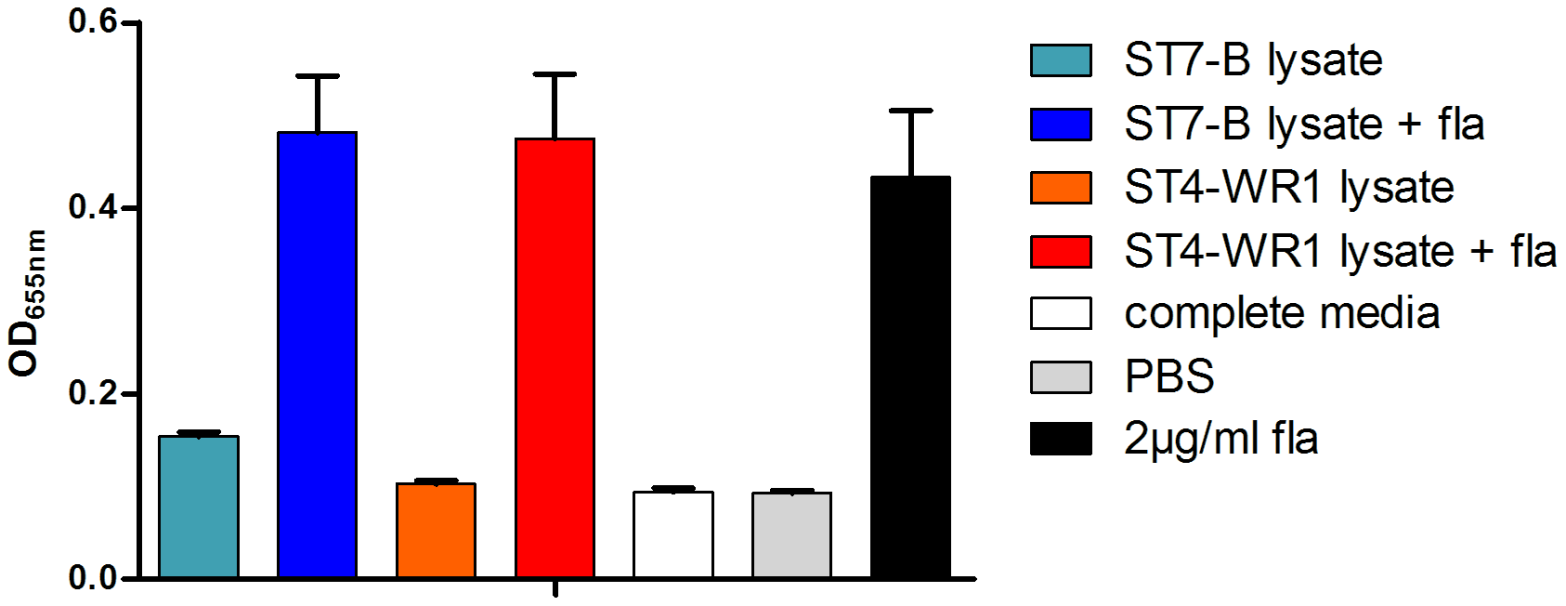
*Blastocystis* effects on flagellin (fla) stimulation of THP1-Blue monocytes. Detection of SEAP activity from cell culture supernatants of THP1-Blue monocytes incubated with various conditions. Parasite lysate was added to THP1-Blue cells at amounts that correspond to a ratio of 1 THP1-Blue cell to 10 parasites. Both *Blastocystis* ST7-B and ST4-WR1 does not significantly modulate fla-induced activation of THP1-Blue monocytes.

The effects of *Blastocystis* ST7-B and ST4-WR1 on NF-κB activation triggered by other TLR ligands were also investigated. Zymosan, a cell wall preparation from the yeast *Saccharomyces cerevisiae* and purified flagellin from the Gram-negative bacteria *Salmonella typhimurium* were used. THP1-Blue cells were incubated with *Blastocystis* WCL, in the presence or absence of a specific ligand. While ST7-B WCL showed an augmenting effect in LPS-mediated NF-κB activation, we observed a dose-dependent inhibition in zymosan-mediated NF-κB activation by both ST7-B and ST4-WR1 WCL (*p*<0.01) (figure 3). However, neither an augmenting nor an inhibiting effect was observed from *Blastocystis* WCL on flagellin-mediated NF-κB activation (figure 4).

## Discussion

To date, there has been no focused investigation on *Blastocystis* spp. and TLRs. Insights into interactions between this enteric parasite and the TLR family of PRRs are likely to provide more in-depth characterization of the pathogenicity of *Blastocystis* spp, given the high prevalence of the parasite in the global human population, the gastrointestinal symptoms associated with it and the role of TLRs in regulating gut immune homeostasis and recognition of luminal pathogens to initiate controlled immune responses [12]. In this study, the THP1-Blue human monocytic cell line was used to investigate *Blastocystis*-host interactions, specifically in the context of TLR and NF-κB activation. This cell line serves as a appropriate surrogate reporter model for experiments to determine if *Blastocystis* spp. activates any TLRs and if it possesses any modulating effects on the activation of certain TLRs by their specific ligands.

Although THP1-Blue naturally expresses all TLRs known to be actively present in human cells, we focused on the three that are relevant for the colonic mucosa. TLR-2, which naturally binds to lipopeptides [12], zymosan [16] and lipoteichoic acid [17], has been shown to be directly involved in the maintenance of intestinal homeostasis [18] and intestinal epithelial barrier integrity [19]. TLR-4 naturally binds to bacterial LPS and is linked to gut inflammation and inflammatory bowel diseases like ulcerative colitis [20], [21]. The colon is the main site of expression for TLR-5 [22], which binds to flagellin of several bacterial species [23]. TLR-2, 4 and 5 have been shown to be expressed on both apical and basolateral sides of the colonic epithelium, depending on the normal healthy and disease states [22], [24].

THP1-Blue cells were incubated with live parasites or parasite components, with or without ligands for TLR-2, 4 and 5. *Blastocystis* spp., in isolation, did not significantly induce NF-κB activation in THP1-Blue cells, as shown by the consistent lack of significant increase in SEAP activity in the culture supernatant following exposure to live parasites or whole cell lysate, thus suggesting for the first time that *Blastocystis* spp. is unable to activate TLRs and downstream signaling cascades leading to NF-κB activation. Previously, there had been no published characterizations on interactions between *Blastocystis* spp. and TLRs. Most known ligands of TLRs are either of bacterial or viral origin, with some protozoan parasites like *Typanosoma cruzi* and *Toxoplasma gondii* activating TLR-2, 4 and TLR-9, 11 respectively [25].

Recently, cysteine proteases of *Blastocystis* ST4-WR1 has been found to mediate IL-8 secretion from human colonic epithelial T84 cells in an NF-κB-dependent manner [26]. Taken together, this suggests that the NF-κB-dependent expression of IL-8 in T84 colonic epithelial cells may be mediated by mechanisms independent of TLRs. Indeed, bacterial pathogens have been observed to induce inflammatory responses via receptors that are not TLRs. For instance, the *Serratia marcesens*-derived protease serralysin induces host inflammatory responses through protease-activated receptor 2 (PAR-2), activating NF-κB and upregulating IL-8 expression [27]. More recently, the same group characterized a novel secreted protease from *Pseudomonas aeruginosa* and found it capable of activating NF-κB through PAR-1, -2 or -4 [28]. In addition to PAR-mediated mechanisms, cyclooxygenase-2-mediated prostaglandin E_2_-dependent modulation of IL-8 production and neutrophil infiltration has been observed in a human fetal intestinal xenograft mouse model used to study *Entamoeba histolytica* infection [29]. Also, toxins from *Clostridium difficile* are observed to target host Rho GTPases and lead to the activation of mitogen-activated protein kinase (MAPK)-activated protein kinase (MK2), which contributes to *Clostridium difficile*-associated epithelial injury and intestinal inflammation [30]. Given the growing complexity in intracellular interactions and signal transduction pathways that drive infection-associated host inflammation, further characterization would have to be carried out to delineate the specific mechanisms associated with *Blastocystis-*induced intestinal inflammatory response.

In experiments between THP1-Blue cells and live *Blastocystis* spp., we observed that live ST4-WR1 parasites were able to dampen LPS-mediated NF-κB activation when THP1-Blue cells were co-cultured with both live ST4-WR1 parasites and LPS. Although this suggests that live ST4-WR1 parasites may modulate immune responses mediated by bacterial components, it was then verified that live ST4-WR1 parasites also significantly dampen SEAP signals from THP1-Blue-depleted culture supernatant collected after LPS pre-treatment of THP1-Blue cells. Nonetheless, *Blastocystis* spp.-mediated modulation of host immune responses has been observed in colonic epithelial cells, in which IL-8 production was observed to be reduced when *Escherichia coli* LPS was co-cultured with the HT-29 colonic epithelial cells along with live *Blastocystis* spp. ST1-NandII parasites [31]. However, tests to directly assess NF-κB activation in human cells after exposure to live parasites, such as measurement of IκB phosphorylation, should be carried out next for definitive verification of data reported in this study.

The significant and direct inhibition of SEAP activity by live parasites presented a limitation in this cell model when exploring TLR stimulation and NF-κB activation in the context of challenge with live *Blastocystis* spp. However, *Blastocystis* spp. WCL was not found to inhibit SEAP activity (data not shown). Hence, in addition to live parasites, we also included in this study, testing for NF-κB activation in THP1-Blue cells using parasite WCL from both ST7-B and ST4-WR1 isolates. Interestingly, previous observations made in experiments exposing THP1-Blue cells to live parasites, in the presence or absence of LPS, were not made again in similar set-ups involving *Blastocystis* spp. WCL. This suggests that the previously observed ST4-WR1 dampening of LPS-mediated NF-κB activation may be mediated only by live ST4-WR1 parasites. Moreover, ST7-B WCL and not ST4-WR1 unexpectedly showed a significant augmenting effect on LPS-mediated THP1-Blue NF-κB activation observed as increased SEAP activity as compared to LPS positive control. This augmenting effect was not observed in experiments involving live ST7-B parasites and we hypothesize that it is mediated by intracellular parasite factors that are not secreted by live parasites and become accessible to host cells when parasite cells undergo lysis and expose intracellular contents to host cells.

Lysis of *Blastocystis* spp. may be mediated physiologically by a variety of different factors. In the human gut, *Blastocystis* spp. is exposed to a gamut of host responses that would effect parasite cell lysis. Mirza *et al.* reported recently that ST4-WR1 and ST7-B are sensitive to nitrosative stress, exhibiting increased apoptotic and necrotic features upon exposure to nitric oxide [32]. Although no study has yet been done on the effects of gut anti-microbial peptides on *Blastocystis* spp., Huang *et al.* had demonstrated the effectiveness of synthetic antimicrobial peptides, analogous to naturally produced frog skin magainin peptides, against *Blastocystis* spp., with total cell destruction and leakage of cellular contents [33]. LL-37, a cathelicidin antimicrobial peptide commonly expressed in the human gut, shares similar structural features with magainin, in that the peptide comprises largely of linear cationic α-helices. In a recent study, Cobo *et al.* showed that another enteric protozoan parasite *Entamoeba histolytica* increased LL-37 expression from colonic epithelial cells and exhibits resistance against LL-37-mediated killing though parasite protease-mediated degradation of LL-37 [34]. It remains to be seen if *Blastocystis* spp. is vulnerable to antimicrobial action of LL-37 and other human antimicrobial peptides like defensins. Considering these examples of innate host responses against gut microbes, we hypothesize that host response-mediated cell lysis does occur in *Blastocystis* spp., which gives rise to parasite cell lysate that would contribute to the observed effects to TLR activation in this study.

Interestingly, there is an increasing body of evidence that suggest TLR4-independent sensing of Gram-negative LPS by host cells [35]. Studies elucidating these novel mechanisms propose that LPS may be delivered into the cytoplasm by cholera toxin B, which in turn activates caspase-11 and trigger IL-1β production [35]. IL-1β is a pro-inflammatory cytokine and has been well-established to be one of several potent inducers that activate NF-κB [36]. However, it is worth noting with caution that the putative internal LPS sensor that is integral to this TLR4-independent mechanism has yet to be identified. Future investigations would also have to be made to determine if elements of *Blastocystis* spp. WCL, if any, are also able to transport LPS across the host cell membrane into the cytoplasm. As such, this remains, at best, a preliminary possibility to explain the augmentation effect observed for LPS-induced NF-κB activation and increased SEAP activity.

The objective of the heat-treated parasite lysate experiments was primarily to begin exploring mechanics behind *Blastocystis* spp.-mediated modulation of ligand-specific TLR stimulation and NF-κB activation. ST7-B-mediated augmentation of LPS-induced TLR4 and NF-κB activation was not affected by the heat treatment. Interestingly, a similar augmentation effect of LPS-induced TLR4 and NF-κB activation became present in ST4-WR1 lysate after heat treatment. This suggests firstly that the components responsible for the augmentation effect in ST7-B lysate were likely to be heat stable; secondly that the counterpart components for the augmentation effect in ST4-WR1 lysate only became active after heat-labile components, possibly proteins, had been deactivated by heat. We hypothesize that denaturation of these heat-labile components in WCL by heating may have altered their structural conformation of these components and rendered them structurally accessible for ligation to other accessory receptors such as scavenger receptors, which may contribute to augmentation of NF-κB activation. To the best of our ability, we have not found in literature any similar observations of a gain in augmentation of host cell NF-κB activation by microbial components after heat treatment.

Taking a broader perspective, findings from recent studies have suggested that a basal activation of TLR-4 in healthy mucosa by selective commensal microbes and factors derived from these organisms confer protection against injury [18], [37] and dampens immunopathological responses against food allergens [38], [39]. One possible hypothesis may be that in the event of a *Blastocystis* spp. infection, parasite subtypes capable of modulating LPS-mediated TLR-4 activation in a positive manner may disrupt this balance of basal TLR-4-dependent signals that contribute towards gut homeostasis, allowing a bystander effect of inflammation to manifest. In addition, TLR-4 has been found to be more highly expressed in intestinal epithelial cells and lamina propria immune cells of inflammatory bowel disease (IBD) patients [12], [22], [40]. Moreover, an emerging but weak association has been reported between higher rates of *Blastocystis* spp. infection patients with ulcerative colitis [41], [42] and IBS [7]. Taken together with previously reported findings of upregulation of pro-inflammatory cytokine gene expression in experimentally infected rats [43]; and increased IL-8 production from human colonic epithelial cells [26], chronic inflammation commonly associated with IBS and IBD may be a result of complex interactions involving LPS and parasite components, activating TLR-4 and other receptors in a concomitant manner.

This augmenting effect from ST7-B WCL for LPS-mediated THP1-Blue NF-κB activation was not observed in the activation of other TLRs by their corresponding ligands. In contrast, both ST4-WR1 and ST7-B WCL showed an inhibitory effect in zymosan-mediated NF-κB activation in THP1-Blue cells, reflected in the decrease in SEAP activity in THP1-Blue culture supernatant in a dose-dependent manner, after exposure *to* Blastocystis spp. WCL of different concentrations. TLR-2 has been implicated to not only have an immune-sensing role against pathogenic bacterial cell wall component, but also functions to enhance ZO-1-associated intestinal epithelial barrier integrity via protein kinase C [19]. Therefore, it is possible that in the event of a *Blastocystis* spp. infection with subtypes that bring about dysregulation of TLR-2 signaling, this effect on host cells may be an alternative mechanism driving previously observed *Blastocystis*-induced epithelial barrier compromise [11].

The disruption of intestinal epithelial barrier integrity by *Blastocystis* spp. may provide TLRs localized beneath the apical surface of the intestinal epithelium access to luminal pathogen-derived ligands. For instance, TLR-5 is the predominant receptor for bacterial flagellin [23], mainly expressed in the basolateral colon [22]. This implicates an accessory role in *Blastocystis* spp. towards bacterial induction of intestinal inflammation, which contributes to an emerging picture of the direct and indirect participation of *Blastocystis* spp. in the disruption of intestinal homeostasis.

In conclusion, our data shows for the first time that *Blastocystis* exhibits pleiotropic effects in the modulation of TLR activation by specific ligands, namely zymosan, LPS and flagellin for TLR-2, TLR-4 and TLR-5 respectively. However, it should be noted that the scope of this study has been confined to 2 isolates of *Blastocystis* spp., which in turn represent only 2 of 14 subtypes identified to date, 9 of which have been identified in humans [44]. *Blastocystis* spp. is known to be genetically diverse [44] and along with other factors like the lack of standardization across studies and diagnostic techniques have led to conflicting reports in support of a role for *Blastocystis* spp. pathogenicity [45]. Indeed, it has recently been demonstrated that high genetic diversity also exists within a subtype [46]. Future experiments should focus on investigations that involve more isolates to represent more subtypes that are clinically relevant to humans; and bringing the study of *Blastocystis* spp.-TLR interactions to the intestinal mucosal context. Through this work, efforts continue to delineate mechanisms that underpin symptoms associated with *Blastocystis* spp. infections and to provide more accurate insights into the pathogenesis of this highly prevalent enteric parasite and how it interacts with gut mucosa. In turn, this information would provide clinicians with a more informed position to diagnose and manage *Blastocystis* spp. infections.
